# Influencing risk factors of voriconazole-induced liver injury in Uygur pediatric patients undergoing allogeneic hematopoietic stem cell transplantation

**DOI:** 10.1186/s12887-024-04625-1

**Published:** 2024-05-03

**Authors:** Ting Zhao, Hui-lan Zhang, Hao Shen, Jie Feng, Ting-ting Wang, Hong-jian Li, Lu-hai Yu

**Affiliations:** 1 Department of Pharmacy, People’s Hospital of Xinjiang Uygur Autonomous Region, Urumqi, 830001 Xinjiang China; 2 Institute of Clinical Pharmacy of Xinjiang Uygur Autonomous Region, People’s Hospital of Xinjiang Uygur Autonomous Region, Urumqi, 830001 Xinjiang China

**Keywords:** *CYP2C19*, HSCT, Liver injury, *UGT1A4*, Voriconazole

## Abstract

**Purpose:**

We aimed to investigated the influencing risk factors of voriconazole-induced liver injury in Uygur pediatric patients undergoing allogeneic hematopoietic stem cell transplantation (HSCT).

**Methods:**

This was a prospective cohort design study. High-performance liquid chromatography-mass spectrometry was employed to monitor voriconazole concentration. First-generation sequencing was performed to detect gene polymorphisms. Indicators of liver function were detected at least once before and after voriconazole therapy.

**Results:**

Forty-one patients were included in this study, among which, 15 patients (36.6%) had voriconazole-induced liver injury. The proportion of voriconazole trough concentration > 5.5 μg·mL^−1^ patients within the DILI group (40.0%) was significantly higher compared to the control group (15.4%) (*p* < 0.05). After administration of voriconazole, the values of ALT (103.3 ± 80.3 U/L) and AST (79.9 ± 60.6 U/L) in the DILI group were higher than that in the control group (24.3 ± 24.8 and 30.4 ± 8.6 U/L) (*p* < 0.05). There was no significant difference between the two groups in genotype and allele frequencies of CYP2C19*2, CYP2C19*3, CYP2C19*17, and *UGT1A4* (rs2011425) (*p* > 0.05).

**Conclusion:**

There was a significant correlation between voriconazole-induced liver injury and voriconazole trough concentration in high-risk Uygur pediatric patients with allogeneic HSCT.

## Background

Invasive fungal infection (IFD) is a dangerous killer for the survival and prognosis of patients with hematopoietic stem cell transplantation [1]. A multicentric prospective study showing that the total incidence of IFD in patients with hematopoietic stem cell transplantation (HSCT) in patients is 7.7%, and the fatality rate of IFD after HSCT is as high as 50% [2]. Voriconazole is the first-line treatment drug for invasive Aspergillus infection and Candida krusei infection. It is now commonly used for the prevention and treatment of fungal infections in patients with hematological diseases during chemotherapy and HSCT [3]. Abnormal liver function is a common adverse reaction during voriconazole treatment, with an incidence of hepatotoxicity ranging from 6.3% to 51.7% [4–6].

Voriconazole is primarily metabolized by *CYP2C19* [7], and, to a lesser extent, *CYP2C9* and *CYP3A4* [8, 9], therefore, CYP450 genetic polymorphisms and CYP-mediated drug interactions are important determinants of interindividual variability of voriconazole exposure [7]. Moreover, both N-oxide and hydroxylation metabolites of voriconazole can be glucuronidated [10]. Bourcier et al. [11] found that uridine diphosphate glucuronyl transferase 1A4 (*UGT1A4*) is the main enzyme involved in the glucuronidation of voriconazole.

Nowadays, the majority of the researches focused on the relationship between hepatotoxicity and polymorphism of cytochrome P450 (*CYP450*), especially the polymorphism of *CYP2C19* [12–14]. However, few studies focused on the relativity between voriconazole-induced liver injury and polymorphism of UGT, especially in Chinese Uygur pediatric patients, leading to difficulties in dose decision-making in such patients in clinical practice.

The purpose of the present study was to evaluate the association between voriconazole-induced liver injury and gene polymorphisms of *CYP2C19* and *UGT1A4* and voriconazole trough concentration in Uygur pediatric patients undergoing allogeneic hematopoietic stem cell transplantation (HSCT).

## Materials and methods

### Pediatric patients

In this prospective observational study, 41 pediatric patients in Chinese Uygur population with allogeneic-HSCT, who received voriconazole therapy were included in the study from November 2020 to May 2023. Inclusion criteria: 2 to 18 years old; pediatric patients with hematological malignancies who underwent allogeneic-HSCT; patients taking voriconazole continuously for more than 3 days. Exclusion criteria: over 18 years of age, those with missing relevant research data, inconsistent blood collection time points, suffering from any diseases that may affect liver function (e.g., fatty liver, viral hepatitis, etc.), and abnormal liver function before voriconazole therapy or combined utilization of other hepatotoxic drugs during voriconazole therapy.

Voriconazole was administered to the prevention and treatment of fungal infections in patients with hematological diseases during chemotherapy and HSCT according to the Chinese guidelines for the diagnosis and treatment of invasive fungal infections in critically ill patients (2017) [15]. TDM of voriconazole for each pediatric patients was conducted at least once during the study period. Voriconazole trough concentration was obtained on day 2 in pediatric patients receiving two loading doses intravenously or orally, on day 6 in pediatric patients who received less than two loading dose, and after 6 doses when the dosage had been adjusted.

The following information was retrieved from pediatric patients's electronic medical record: demographic data, clinical diagnosis, the dosage regimen of voriconazole (dosage form, dosage, frequency, administration route), plasma trough concentration data, and concomitant medications during voriconazole therapy and indicators of liver function. Indicators of liver function, including alanine aminotransferase (ALT), aspartate aminotransferase (AST), total bilirubin (TBIL), creatinine (Cr), and blood urea nitrogen (BUN) were detected at least once before voriconazole therapy, and at least once during voriconazole therapy.

According to the National Cancer Institute Common Terminology Criteria for Adverse Events version 4.0 [5], drug-induced liver injury (DILI) is defined as the level of at least one indicator aforementioned being higher than the upper limit of normal range after the initiation of voriconazole therapy. Causality between liver injury and voriconazole therapy was assessed using the the standardized Roussel Uclaf Causality Assessment Method.

All patients enrolled in this study were classified into DILI case group or control group. This study was granted approval by the Ethics Committee of People’s Hospital of Xinjiang Uygur Autonomous Region, Xinjiang, China (ethical approval number: XJS2021111809). The patients and the parents of all pediatric patients signed an informed consent form.

### Therapeutic drug monitoring of voriconazole

For drug concentrations assays, 1–2 mL of venous blood was obtained from each patient just before the morning voriconazole dose was administered (approximately 12 h after the evening dose; trough concentration).Voriconazole plasma trough concentration was measured by a validated high-performance liquid chromatography-tandem mass spectrometry (LC–MS/MS) method. The extraction process was initiated by adding 300 µL of organic deproteinization solution (Abbott Laboratories, Shanghai, China) to 50 µL of plasma sample. The upper organic layer was collected for voriconazole plasma concentration determination.

Liquid chromatography: The column was Waters ACQUITY UPLC® BEH (3.0 × 50 mm, 2.5 μm) column (Waters Technology Co., LTD. Shanghai, China). The column was eluted with a gradient elution program using a mobile phase composed of 0.01% formic acid in water (mobile phase A) and 0.01% formic acid in acetonitrile (mobile phase B). The column temperature was 40 ºC. The injection volume was 2 µL. Mass spectrometry: AB SCIEX TRIPLE QUAD ™ 4500MD (AB Sciex, CA, USA) was employed with an electrospray ionization source (ESI) and positive polarity. The source parameters included curtain gas of 40 psi, ion spray voltage of 5500 V, an ion source temperature of 500°C, and medium collision gas. Quantification was performed by multiple- reaction-monitoring of the transitions at m/z 350.2–127.1 for voriconazole and m/z 353.2–130.1 for the IS.

### Genotyping

**Fig. 1:**
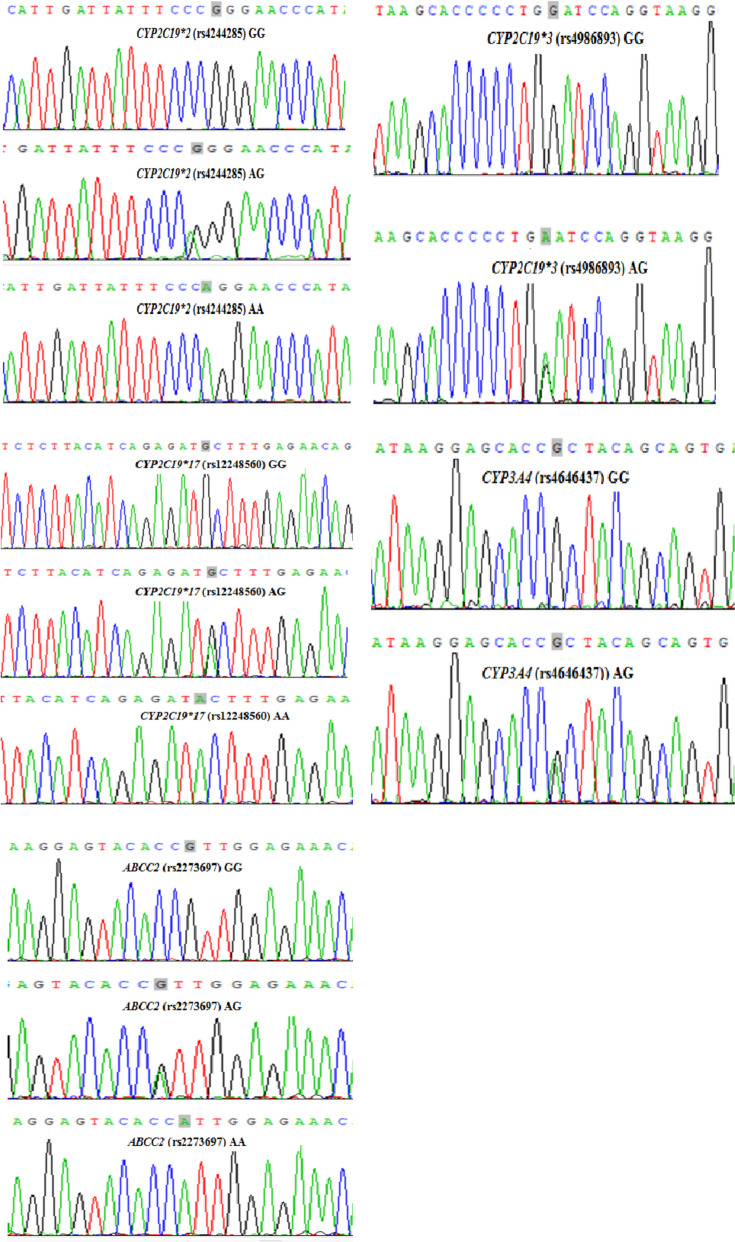
PCR amplifications of the rs4244285, rs4986893, rs12248560, rs4646437, and rs2273697 loci, digested by BanI. M: represents marker

Total genomic DNA was extracted from peripheral leukocytes using the Tiangen kit (Tiangen Biochemical Technology Co., Ltd., Beijing, China). The CYP2C19*2 (681G > A, rs4244285), CYP2C19*3 (636G > A, rs4986893), CYP2C19*17 (-806C > T, rs12248560), and *UGT1A4* (142 T > G, rs2011425) gene polymorphisms were genotyped using a PCR assay using Big Dye (Applied Sanger Sequencing Technologies, Hangzhou, China). Through PCR amplification, agarose electrophoresis detection, gel recovery and other steps, the amplified PCR products were detected and purified, and the PCR products were sequenced using the 3730XL sequencer produced by ABI Company in the United States. Table 1 provides detailed information on the primers and the target fragment sizes. Figure 1 presents the results of DNA sequencing analysis for each genotype.

**Table 1:** Sequences of the primers used in the study

SNP	Forward primer	Reverse primer
CYP2C19*2 (681G > A, rs4244285)	CAACCAGAGCTTGGCATATTGTATCT	CGAGGGTTGTTGATGTCCATCGAT
CYP2C19*3 (636G > A, rs4986893)	GCTGTGCTCCCTGCAATGTGATCT	GGCTAGAAGCCTGATCTATATTGGGA
CYP2C19*17 (-806C > T, rs12248560)	GCCCTTAGCACCAAATTCTC	ACACGTGAAGGCAGGAATTG
*UGT1A4* (142 T > G, rs2011425)	GGAAACAAATGTAGCAGGCA	ACCCTTGAGTGTAGCCCAGC

*SNP* single nucleotide polymorphism, *bp* base pair

The gene encoding *CYP2C19* is highly polymorphic, with more than 34 variant alleles identified (http://www.cypalleles.ki.se) [16]. According to nomenclature by the Clinical Pharmacogenetics Implementation Consortium [17], patients with the *1/*17 genotype were classified as rapid metabolizer (RM), and those with *17/*17 genotype were classified as ultrarapid metabolizer (UM). Patients with one copy of a *2 or a *3 allele (e.g. *1/*2, *1/*3, *2/*17) were assigned the intermediate metabolizer (IM) phenotype, and carriers of two copies (e.g. *2/*2) were assigned the poor metabolizer (PM) phenotype. The normal metabolizer (NM) phenotype was assigned by default to patients without a *2, *3, or *17 allele.

### Statistical analysis

Statistical analyses were carried out using SPSS (version 4.0.100.1124; Chicago, IL, Beijing, China). A *p*-value of < 0.05 was considered to be statistically significant. Value data of the normal distribution was expressed as Mean and standard deviation (SD), comparisons between groups were performed with independent sample *t*-test and single factor analysis of variance (ANOVA). The Hardy–Weinberg equilibrium was tested for each polymorphism by the chi-square (χ^2^) analysis. Categorical data was expressed as frequency and percentage, using chi-square test or Fisher's exact test. Spearman correlation was used to test the relationship between variables.

## Results

### Pediatric patients

In this study, a total of 58 pediatric patients with abnormal liver function were collected. Six patients had a history of hepatitis A or hepatitis B, and the liver function of 11 patients was abnormal before treatment with voriconazole. At last, 41 patients were included in this study. The 41 patients were divided into two groups based on whether the liver injury was caused by voriconazole. This included the DILI group (*n* = 15) and control group (*n* = 26). In the DILI group, 15 patients (36.6%) had voriconazole-induced liver injury, and causality assessment result was probable (score 6–7) based on the Roussel Uclaf Causality Assessment Method scale. In the control group, 26 patients (63.4%) had the liver injury was caused by other causes (eg, hepatitis and anti-infective drugs, etc.).

Overall, 68.3% of the patients were male (*n* = 28). The voriconazole maintenance dose ranged from 100 to 400 mg per day. At the last follow-up, 41 patients received cyclosporin A combination therapy, 11 patients received liver enzymes inhibitors combination therapy, and 2 patients received liver drug enzymes inducers combination therapy. The clinical and demographic characteristics of all enrolled pediatric patients are summarized in Table 2.

**Table 2:** The clinical information of populations (the mean ± standard deviations)

Variables	Overall (*n* = 41)	DILI group (*n* = 15)	Control group (*n* = 26)	χ^2^/*t*	*p*-value
Age (years)	10.2 ± 3.0	10.3 ± 2.7	10.1 ± 3.2	0.152	0.880
Gender, n (%)					
Males	28 (68.3)	11 (73.3)	17 (65.4)	1.496	0.221
Female	13 (31.7)	4 (26.7)	9 (34.6)		
Disease status					
Leukemia	21 (51.2)	7 (46.7)	14 (53.8)	0.980	0.322
Aplastic anemia	13 (31.7)	5 (33.3)	8 (30.8)	0.092	0.762
Bone marrow abnormalities	5 (12.2)	2 (13.3)	3 (11.5)	0.058	0.810
Lymphoma	2 (4.9)	1 (6.7)	1 (3.9)	0.866	0.352
Body mass index (kg/m^2^)	15.0 ± 2.8	15.0 ± 2.5	15.1 ± 2.7	-0.036	0.971
Route of administration, n (%)					
Oral administration	34 (82.9)	11 (73.3)	23 (88.5)	7.345	0.007*
Intravenous administration	7 (17.1)	4 (26.7)	3 (11.5)		
Voriconazole dose (mg·kg^−1^·d^−1^)	14.3 ± 4.3	14.0 ± 3.1	14.5 ± 5.2	-0.404	0.689
Voriconazole concentration (μg·mL^−1^)	3.8 ± 2.6	5.5 ± 2.2	2.8 ± 1.8	3.577	< 0.001**
< 1.0 μg·mL^−1^, n (%)	3 (7.3)	0 (0)	3 (11.5)	12.636	< 0.001**
1.0–5.5 μg·mL^−1^, n (%)	28 (68.3)	9 (60.0)	19 (73.1)	3.793	0.051
> 5.5 μg·mL^−1^, n (%)	10 (24.4)	6 (40.0)	4 (15.4)	15.674	< 0.001**
Before voriconazole					
ALT (U/L)	19.1 ± 16.9	24.3 ± 24.8	16.1 ± 9.3	1.222	0.239
AST (U/L)	28.2 ± 9.6	30.4 ± 8.6	27.0 ± 10.0	1.139	0.263
TBIL (μmol·L^−1^)	10.2 ± 4.3	10.1 ± 4.6	10.3 ± 4.2	-0.140	0.890
Scr (mmol·L^−1^)	42.3 ± 14.5	42.4 ± 13.4	41.2 ± 15.3	0.037	0.970
BUN (mmol·L^−1^)	5.4 ± 2.8	6.0 ± 3.6	5.0 ± 2.3	0.978	0.339
After voriconazole					
ALT (U/L)	56.2 ± 63.9	103.3 ± 80.3	29.0 ± 28.5	4.229	< 0.001**
AST (U/L)	52.8 ± 44.4	79.9 ± 60.6	37.1 ± 19.9	2.657	0.017*
TBIL (μmol·L^−1^)	12.2 ± 9.7	14.2 ± 10.0	11.1 ± 9.6	0.991	0.330
Scr (mmol·L^−1^)	41.4 ± 14.5	41.3 ± 14.4	41.5 ± 14.9	-0.056	0.956
BUN (mmol·L^−1^)	6.2 ± 3.2	6.4 ± 4.3	6.0 ± 2.4	0.411	0.683
Co-medication					
Cyclosporin A	39 (95.1)	15 (100.0)	24 (92.3)	8.333	0.004*
Acyclovir	34 (82.9)	12 (80.0)	22 (84.6)	0.866	0.352
Glucocorticoids	18 (43.9)	9 (60.0)	9 (34.6)	12.531	< 0.001**
Anti-infective drugs	18 (43.9)	6 (40.0)	12 (46.1)	0.734	0.391
Hepatoprotective drugs	17 (41.5)	7 (46.7)	10 (38.5)	1.444	0.230
Co-medication					
Liver drug enzyme inhibitor	9 (21.9)	4 (26.7)	5 (19.2)	1.807	0.179
Liver drug enzyme inducer	18 (43.9)	9 (60.0)	9 (34.6)	12.531	< 0.001**

*DILI* Drug-induced liver injury, *TBIL* Total bilirubin, *ALT* alanine transaminase, *AST* aspartate transaminase, *TB* total bilirubin, *Scr* serum creatinine, *BUN* blood urea nitrogen

^*^ *p*-value < 0.05

^**^ *p*-value < 0.001

There was no significant difference in age, gender, BMI, voriconazole dose, and disease status between the DILI group or control group (*p* > 0.05). The proportion of male patients (73.3% and 65.4%) in the DILI group and the control group was higher than that in female patients (26.7% and 34.6%) (*p* > 0.05). The diagnosis of diseases in patients taking voriconazole mainly included leukemia (51.2%), aplastic anemia (31.7%), bone marrow abnormalities (12.2%), lymphoma (4.9%). There was no difference in disease status between the DILI group or control group (*p* > 0.05). There was no significant difference in the voriconazole dose between the DILI group (14.0 ± 3.1 mg·kg^−1^·d^−1^) and the control group (14.5 ± 5.2 mg·kg^−1^·d^−1^) (*p* > 0.05).

### Plasma voriconazole concentrations and co-medication

Our data showed that there was a significant difference in route of administration, voriconazole concentration, and co-medication between the DILI group or control group (*p* < 0.05) (Table 2). The proportion of oral administration patients within the control group (88.5%) was significantly higher compared to the DILI group (73.3%) (*p* < 0.05). The proportion of voriconazole trough concentration > 5.5 μg·mL^−1^ patients within the DILI group (40.0%) was significantly higher compared to the control group (15.4%) (*p* < 0.05).

The proportion of voriconazole in combination with cyclosporine A and glucocorticoids patients within the DILI group (100% and 60.0%) was significantly higher compared to the control group (92.3% and 34.6%) (*p* < 0.05). The proportion of voriconazole in combination with liver drug enzyme inducer patients within the DILI group (60.0%) was significantly higher compared to the control group (34.6%) (*p* < 0.05).

**Fig. 2:**
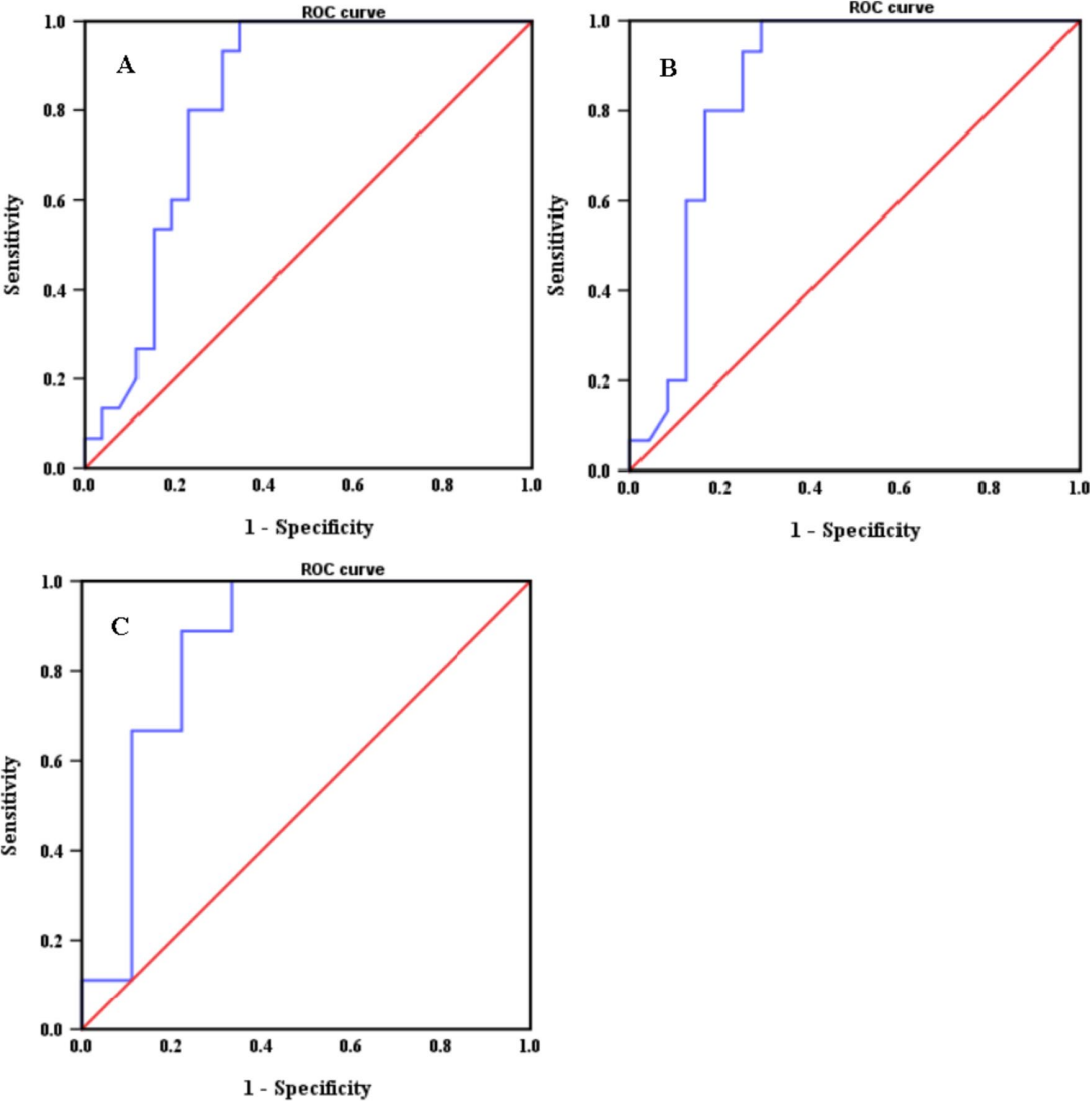
Receiver operating characteristic (ROC) curve of plasma voriconazole concentration in relation to voriconazole-induced liver injury (A. voriconazole; B. voriconazole and cyclosporine A were combined; C. voriconazole and glucocorticoids were combined)

An ROC curve relating the DILI and plasma voriconazole concentration was constructed (Fig. 2).The accuracy of plasma voriconazole concentration in predicting DILI was high {[area under the diagnostic curve (AUC) (95% CI)] = 0.819 (0.690 – 0.949)}. When the plasma voriconazole concentration ≥ 3.6 μg·mL^−1^, the likelihood of DILI is higher (the sensitivity and specialties were 0.933 and 0.308, respectively). Meanwhile, when voriconazole and cyclosporine A were combined, the probability of DILI was higher when the plasma voriconazole concentration ≥ 3.0 μg·mL^−1^ (the sensitivity and specialties were 0.933 and 0.250, respectively). When voriconazole and glucocorticoids were combined, the probability of DILI was higher when the plasma voriconazole concentration ≥ 3.3 μg·mL^−1^ (the sensitivity and specialties were 0.889 and 0.222, respectively).

### Voriconazole-induced liver injury

Before the voriconazole therapy, ALT, AST, TBIL, Scr and BUM of all patients were within the normal range, and there was no significant difference in these indicators between the the two groups (*p* > 0.05). After administration of voriconazole, the values of ALT (103.3 ± 80.3 U/L) and AST (79.9 ± 60.6 U/L) in the DILI group were higher than that in the control group (24.3 ± 24.8 U/L and 30.4 ± 8.6 U/L) (*p* < 0.05; Table 3).

**Table 3:** Changes of liver and kidney function in children before and after voriconazole (mean ± standard deviation)

Time	Liver function			Kidney function	
	ALT (U/L)	AST (U/L)	TBIL (μmol·L^−1^)	Scr (mmol·L^−1^)	BUN (mmol·L^−1^)
Before voriconazole	24.3 ± 24.8	30.4 ± 8.6	10.1 ± 4.6	42.4 ± 13.4	6.0 ± 3.6
After voriconazole	103.3 ± 80.3	79.9 ± 60.6	14.2 ± 10.0	41.3 ± 14.4	6.4 ± 4.3
*t*-value	-3.641	-3.133	-1.443	0.216	-2.264
*p*-value	< 0.05*	< 0.05*	0.160	0.831	0.794

*ALT* alanine transaminase, *AST* aspartate transaminase, *TB* total bilirubin, *Scr* serum creatinine, *BUN* blood urea nitrogen

^*^ *p*-value < 0.05

### Genotype and allele frequencies

All gene polymorphisms studied followed Hardy–Weinberg equilibrium in the DILI group or control group patients (*p* > 0.05), which indicated that the research subjects were representative of entire group. The genotype and allele frequencies of the two groups are shown in Table 4. There was no significant difference between the two groups in genotype and allele frequencies of CYP2C19*2, CYP2C19*3, CYP2C19*17, and *UGT1A4* (*p* > 0.05). We also analyzed the relationship of DILI and phenotype of *CYP2C19*, but there was no statistical difference.

**Table 4:** The relationship of voriconazole-induced liver injury and polymorphism of CYP2C19*2, *3, *17, UGT*1A4*

SNP	Genotype	DILI group (*n* = 15)	Control group (*n* = 26)	χ^2^	OR value (95% CI)	*p*-value
CYP2C19*2 (681G > A, rs4244285)	GG	11 (73.3)	16 (61.5)	3.073	1.701 (0.937–3.087)	0.080
	GA	3 (20.0)	8 (30.8)	3.185	0.556 (0.291–1.064)	0.074
	AA	1 (6.7)	2 (7.7)	0.072	0.866 (0.302–2.485)	0.788
	G	25 (83.3)	40 (76.9)	1.125	1.458 (0.725–2.935)	0.289
	A	5 (16.7)	12 (23.1)			
CYP2C19*3 (636G > A, rs4986893)	GG	14 (93.3)	25 (96.1)	0.866	0.554 (0.157–1.954)	0.352
	GA	1 (6.7)	1 (3.9)			
	G	29 (96.7)	51 (98.1)	0.205	0.660 (0.108–4.036)	0.651
	A	1 (3.3)	1 (1.9)			
CYP2C19*17 (-806C > T, rs12248560)	CC	12 (80.0)	20 (76.9)	0.267	1.195 (0.608–2.349)	0.606
	CT	3 (20.0)	6 (23.1)			
	C	27 (90.0)	46 (88.5)	0.182	1.213 (0.499–2.9352)	0.669
	T	3 (10.0)	6 (11.5)			
*CYP2C19* metabolizers	EM	8 (53.3)	11 (42.3)	2.426	1.557 (0.891–2.722)	0.119
	IM	4 (26.7)	9 (34.6)	1.496	0.687 (0.376–1.256)	0.221
	PM	1 (6.7)	2 (7.7)	0.072	0.866 (0.302–2.485)	0.788
	UM	2 (13.3)	4 (15.4)	0.166	0.847 (0.380–1.886)	0.684
*UGT1A4* (142 T > G, rs2011425)	TT	11 (73.3)	17 (65.4)	1.496	1.456 (0.796–2.661)	0.221
	TG	4 (26.7)	9 (34.6)			
	T	26 (86.7)	43 (82.7)	0.627	1.371 (0.627–2.997)	0.428
	G	4 (13.3)	9 (17.3)			

*DILI* Drug-induced liver injury, *OR* Odds ratio, *SNP* Single nucleotide polymorphism, *PM* poor metabolizers, *IM* intermediate metabolizers, *EM* normal metabolizers, *UM* ultra-fast metabolizers

### Influence of CYP2C19 and UGT1A4 genotype on plasma voriconazole concentrations

Among the included cases, three patients had voriconazole trough concentration < 1.0 μg·mL^−1^, ten patients had voriconazole trough concentration > 5.5 μg·mL^−1^ (beyond the recommended treatment range). The results are shown in Table 2.

We discovered that the CYP2C19*2, CYP2C19*3, CYP2C19*17, and *UGT1A4* polymorphisms had no significant influence on plasma voriconazole concentration and CD ratio (*p* > 0.05), as shown in Table 5. The results showed that the voriconazole trough concentrations and CD ratio of pediatric patients with different *CYP2C19* metabolic types were no significantly different (*p* > 0.05).

**Table 5:** The effect of genotype on voriconazole plasma concentrations and concentration-to-dose ratio in DILI group (*n* = 15)

Single nucleotide polymorphism	Genotype	Number (%)	Plasma concentration (μg·mL^−1^)	*p*-value	CD (μg·mL^−1^·kg·mg^−1^)	*p*-value
*CYP2C19* metabolizers	EM	8 (53.3)	5.0 ± 2.8	0.622	0.4 ± 0.2	0.680
	IM	4 (26.7)	6.6 ± 1.2		0.5 ± 0.1	
	PM	1 (6.7)	6.6 ± 0.0		0.5 ± 0.0	
	UM	2 (13.3)	4.5 ± 0.8		0.3 ± 0.1	
	EM compared to IM	8 (53.3) vs 4 (26.7)	5.0 ± 2.8 vs 6.6 ± 1.2	0.284	0.4 ± 0.2 vs 0.5 ± 0.1	0.321
	EM compared to PM	8 (53.3) vs 1 (6.7)	5.0 ± 2.8 vs 6.6 ± 0.0	-	0.4 ± 0.2 vs 0.5 ± 0.0	-
	EM compared to UM	8 (53.3) vs 2 (13.3)	5.0 ± 2.8 vs 4.5 ± 0.8	0.810	0.4 ± 0.2 vs 0.3 ± 0.1	0.768
*UGT1A4* (142 T > G, rs2011425)	TT	11 (73.3)	5.6 ± 2.2	0.649	0.4 ± 0.2	0.575
	TG	4 (26.7)	5.0 ± 2.5		0.4 ± 0.2	

*CD* concentration-to-dose ratio, *PM* poor metabolizers, *IM* intermediate metabolizers, *EM* normal metabolizers, *UM* ultra-fast metabolizers

## Discussion

Voriconazole is currently widely used clinically in our hospital, especially in the transplant ward of the Department of Hematology. The plasma concentration of voriconazole has a narrow therapeutic window (1.0–5.5 μg·mL^−1^) [18]. Relevant studies have shown that voriconazole plasma trough concentration > 2 μg·mL^−1^ is related to clinical efficacy, and when plasma trough concentration < 1 μg·mL^−1^, the clinical response rate is lower, and the treatment effect is poor [19]. In terms of adverse reactions, voriconazole trough concentration > 5 μg·mL^−1^ is likely to cause drug toxicity, and > 6 μg·mL^−1^ is likely to cause an increase in AST or ALT values [20].

In this study, the trough concentrations of voriconazole were determined in 41 high-risk children with allogeneic HSCT, among which three patients had voriconazole trough plasma concentrations < 1.0 μg·mL^−1^, 10 patients had voriconazole trough concentrations > 6 μg·mL^−1^ (among them, six patients had a significant increase in ALT or AST values after using voriconazole). After administration of voriconazole, the values of ALT and AST in the DILI group were higher than that in the control group (*p* < 0.05). Moreover, our data showed that the proportion of voriconazole trough concentration > 5.5 μg·mL^−1^ patients within the DILI group (40.0%) was significantly higher compared to the control group (15.4%) (*p* < 0.05). Therefore, in order to avoid high-concentration toxicity and low-concentration treatment failure, it is very necessary to control the plasma trough concentration of voriconazole within the effective concentration range. However, the "safe" concentration range (1.0–5.5 μg·mL^−1^) is not necessarily safe for all patients, and there are still latent risks.

Meanwhile, the patient developed liver impairment 15–20 days after taking voriconazole. Among them, ten patients developed hepatotoxicity within 20 days and five patients developed hepatotoxicity within 15 days of voriconazole administration, suggesting that liver function should be closely monitored within 20 days before voriconazole use. ALT and AST are not transiently elevated in patients with hepatic impairment and persist for at least seven days, and decrease to normal after 15 to 20 days of hepatoprotective therapy.

With the rapid development of molecular genetics and pharmacology, individualized treatment based on gene polymorphisms has become the trend and development direction of precision medicine. Drug metabolism is responsible for converting drugs to compounds that are more water soluble and easily excreted but may also be involved in the process of converting drugs into toxic metabolites [21]. The drug metabolizing enzymes mainly involved in this process are the phase 1 enzymes (*CYP450*) and the phase 2 enzymes (N-Acetyltransferases, UGT, et al.) [4]. At present, the association between DILI and genetic polymorphisms relevant to drug metabolism, such as *CYP450*, *UGT*, N-acetyltransferases and glutathione S-transferases has been reported [22–24]. Daly AK et al. [22] showed that the polymorphism of *CYP2C9* and *CYP2C8* was associated with diclofenac-induced liver injury. Chan SL et al. [23] study have found that isoniazid- induced liver injury is not only associated with the gene polymorphism of phase 1 metabolic enzyme *CYP2E1*. The polymorphism of *UGT* was reported to be associated with certain drugs inducing liver injury including antituberculotic, nonsteroidal anti-inflammatory drugs and so on [25, 26].

However, there were few studies on the relationship between gene polymorphism of other metabolic enzyme and voriconazole-induced liver injury [14, 27, 28]. Especially the study referring to the association between *UGT1A4* and voriconazole-induced liver injury was rare. Thus, we investigated the association of *CYP2C19* and *UGT1A4* polymorphisms with voriconazole-induced liver injury. Our results showed that there was no significant correlation between voriconazole-induced liver injury and gene polymorphisms of *CYP2C19* and *UGT1A4*. Therefore, in clinical use of voriconazole, we suggest that the biochemical indicators of liver function should be monitored, so as to find liver injury as soon as possible. And based on the grade of liver injury, the therapeutic regimen could be adjusted, for example, adding hepatoprotective drugs, stoping medicine in severe cases or exchanging to other antifungal drugs.

Our study had some limitations. First, pediatric patients present with limitations such as poor compliance to drug dosing. Second, regional differences, the sample size, and patient evaluation of their own condition also limited this study. Third, considering that there are multiple factors that can affect voriconazole pharmacokinetics and treatment outcomes, there is always a possibility of confounders, including other SNPs. Other genotypes, such as *CYP2C9* and *CYP3A4*, were also considered, all of which were shown to be not significantly associated with voriconazole-induced hepatic impairment. Next, we need to expand the sample size for more extensive genetic screening, with the aim of revealing other potential factors influencing voriconazole metabolism and toxicity, while further explore the mechanism of voriconazole-induced liver injury.

## Conclusion

In conclusion, it has certain practical significance to guide the use of voriconazole in high-risk children with allogeneic HSCT by monitoring the plasma trough concentration of voriconazole. Next, we need to expand the sample size to further explore the mechanism of voriconazole-induced liver injury.

## Data Availability

All the data generated and/or analyzed during this study available from the corresponding author on reasonable request. The datasets generated and/or analysed during the current study are available in the European Variation Archive (EVA) DATE repository, (Submission ID:#718482:).
